# Risk Associated with Malaria Infection in Tihama Qahtan, Aseer Region, Kingdom of Saudi Arabia: 2006-2007

**DOI:** 10.4172/2470-6965/1000144

**Published:** 2016-04-30

**Authors:** AM Alshahrani, TM Abdelgader, M Mohya, S Jubran, AMO Abdoon, AA Daffalla, A Babiker, D Kyalo, AM Noor, MH Al-Zahrani, RW Snow

**Affiliations:** 1 Vector Control Administration, Aseer Health Affairs Directorate, Abha, Kingdom of Saudi Arabia; 2 Aseer General Directorate of Health Affairs, Abha, Kingdom of Saudi Arabia; 3 Public Health Directorate, Ministry of Health, Riyadh, Kingdom of Saudi Arabia; 4 Deanship of Scientific Research, Jazan University, Kingdom of Saudi Arabia; 5 Tropical Medicine Research Institute, National Centre for Research, Sudan; 6 Spatial Health Metrics Group, Kenya Medical Research Institute-Wellcome Trust Research Programme, Nairobi, Kenya; 7 Centre for Tropical Medicine & Global Health, Nuffield Department of Clinical Medicine, University of Oxford, Oxford, United Kingdom; 8 National Malaria Control Programme, Ministry of Health, Riyadh, Kingdom of Saudi Arabia

**Keywords:** Malaria epidemiology, Risk-factors, *Plasmodium falciparum*, Elimination, Case-detection, Aseer, Tihama Qahtan, Saudi Arabia

## Abstract

**Introduction:**

Since 2004, the Kingdom of Saudi Arabia has pursued a policy of malaria elimination. The distribution of malaria at this time was constrained to regions located in the South Western part of the country. The present study aimed to understand the risk of malaria infection and factors associated with these events between March 2006 and August 2007 in one part of Aseer region.

**Methods:**

The study was carried out in Tihama Qahtan area in the far southeastern part of Aseer, historically the most malaria endemic area of this region. The area covers 54 villages served by three primary health care centres (Wadi Alhayah, Alfarsha and Albuqaa). Malaria cases were detected using passive case detection (PCD) at the three health centres for 18 months from March 2006, each positive case was investigated using patient and household level enquiries. In addition, four cross-sectional surveys in 12 villages were undertaken using rapid diagnostic tests within the catchments of each health centre coinciding with malaria transmission seasons.

**Results:**

Among 1840 individuals examined in the PCD survey, 49 (2.7%) were positive for malaria, most were *Plasmodium falciparum* cases and one was a *P. vivax* case. The majority of these infections were likely to have been acquired outside of the area and represent imported cases, including those from the neighboring region of Jazan. Among the 18 locally acquired cases, the majority were adult males who slept outdoors. 3623 individuals were screened during the cross-sectional surveys, 16 (0.44%) were positive and infections only detected during peak, potential transmission periods.

**Conclusion:**

There was evidence of local malaria transmission in the Tihama Qahtan area in 2006-2007, however prevalence and incidence of new infections was very low, making the future ambitions of elimination biologically feasible. The constant source of imported infections must be considered in the area’s elimination ambitions, alongside strong behavioural community messages about sleeping outdoors unprotected and travel to malaria endemic areas outside the region.

## Introduction

Since the epidemics of malaria in the South Western regions of the Kingdom of Saudi Arabia in the late 1990s early 2000s [1–3], the Ministry of Health has pursued a policy of malaria elimination [3,4]. Efforts, since 2004, have concentrated on the remaining Afrotropical malaria eco-epidemiological regions of Aseer and Jazan, where *Plasmodium falciparum* transmission is predominantly maintained by *Anopheles arabiensis* [5]. These administratively autonomous regions border Yemen, which despite agreements on cross-border control in 2007 [6], continues to pose threats to sustaining the reduction of locally transmitted infections due to large population movements across the border from endemic parts of Yemen [2,4,7].

The worst affected area of Aseer region is the Tihama Qahtan lowlands area, described through community-based malaria surveys as hypo-endemic during the 1970s [8]. Malaria in this area was a predominantly paediatric clinical problem during the late 1980s and early 1990s, suggesting stable endemicity leading to acquired functional immunity [9]. Parasite transmission in this area was acutely seasonal, with peaks in disease between February and April [9], following the rains and expansion of *An. arabiensis* populations [10,11]. The Tihama lowlands were largely remote and inaccessible during the 1980s and epidemics were documented in the early-1990s [12]. However, between 1996 and 2005 malaria incidence began to decline [1,2]. By 2005, the total number of locally acquired *P. falciparum* cases detected in the region was only 18, compared to over 700 cases five years earlier.

Identification of the risk factors associated with developing malaria infections and clinical disease is an important step in the process of evaluating the needs of elimination ambitions. The present study describes the characteristics of malaria cases detected at three Primary Health Care Centres (PHCC) and cross-sectional surveys in the Tihama Qahtan area of Aseer region.

## Methods

### Study area

**Figure 1:**
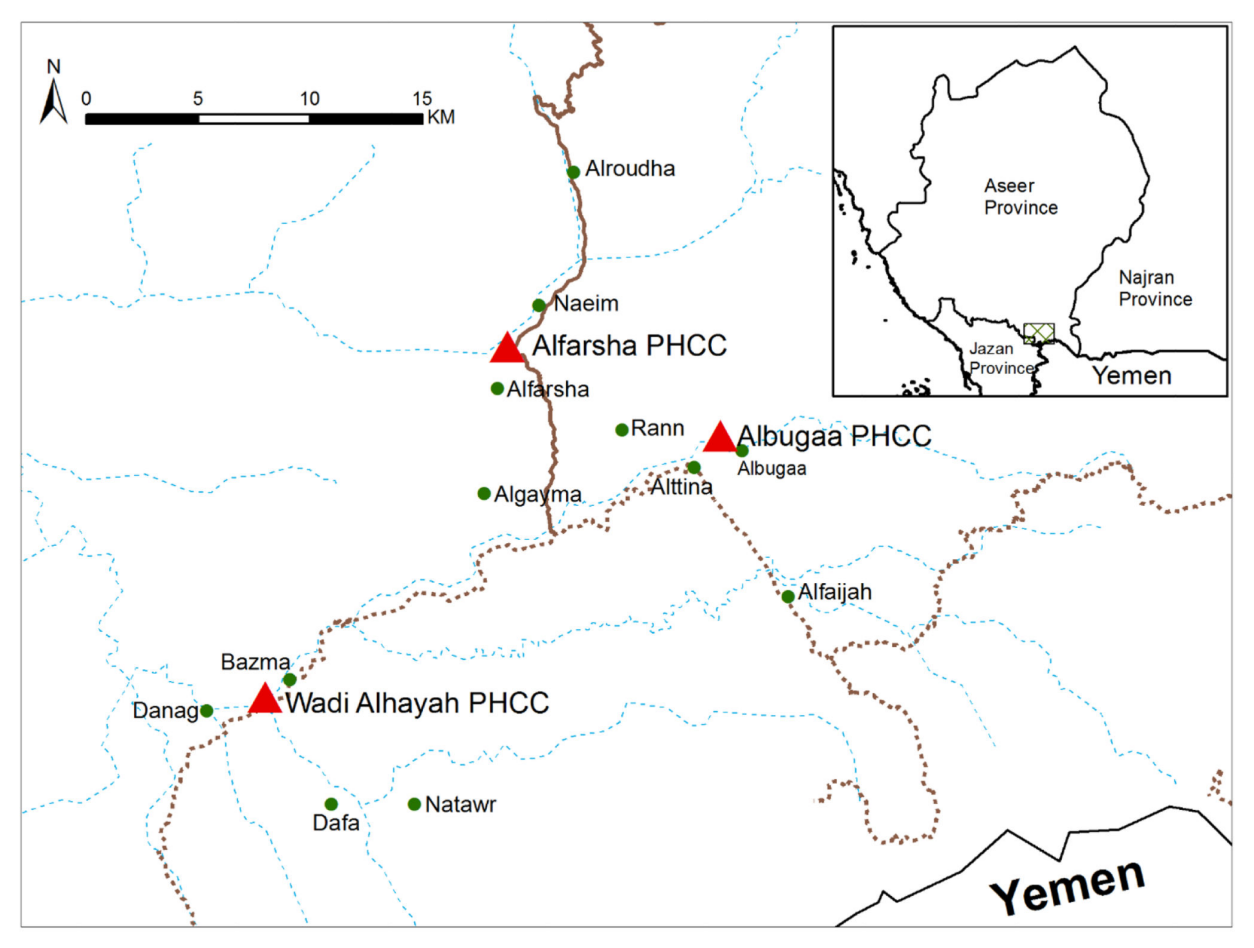
Map of the Tihama Qahtan study area showing the location of the three PHCCs (Red triangles) and the 12 sampled villages (Green dots) used during cross-sectional surveys. Insert map shows location of Aseer and study area in relation to region and neighboring regions. Map digitized using ARCGIS version 10.1, ESRI, USA, from maps provided by MoH, maps provided in [16] and other online digital GIS platforms [17,18].

The study was carried out in the Tihama Qahtan area with an estimated population of about 18,000 living in 54 villages [13], sharing the common border with Jazan Region and the Tihama plains of the Republic of Yemen, considered the highest malaria endemic area of Yemen [14]. The Tihama Qahtan area ranges from sea level to 2100 metres above sea level and is coursed by permanently running waters from 13 main wadis that lead to the largest Wadi Baish. Floods, as a result of heavy rains at the top of the surrounding mountains, start to flush these wadis from late March until May and from July to September [15]. The area is rich in vegetation covering the highlands and the foothills. The population of this area are rural, traditional Tihama tribal communities who are animal herders, with cash incomes from agriculture including fruits. Prior to the mid-2000s, the most common health threats for young children were malaria, anaemia, malnutrition, diarrheal diseases, brucellosis, and bites from scorpions and snakes (MOH Aseer, unpublished data). The study area was divided into three parts, each served by a PHCC, Alfarsha, Albugaa and Wadi Alhayah, providing basic health services and served as the base for malaria diagnostics, treatment and community-based malaria control operations (Figure 1).

### Passive case detection (PCD)

All patients attending the three outpatient clinics of Alfarsha, Albugaa and Wadi Alhayah PHCCs during the period March 2006 to August 2007, complaining of a febrile illness and suspected of being malaria were included in the study. Thick and thin blood films were prepared at the laboratory directly from a finger-prick blood sample. The history of illness, present symptoms and duration, body temperature, residence, age, gender, nationality, and blood test results including parasite species were recorded on a case record form developed specially for the study. All microscopists in the health centres were employed and trained by professional staff from Ministry of Health, all clinical examinations were undertaken by physicians.

The home of each positive malaria case detected during the PCD survey was visited within 48 hours of the notification. At each household, details were recorded on the travel history, sleeping patterns (indoors versus outdoors), presence of migrant laborers in close proximity to the household, type of housing, number of household inhabitants, sleeping habits, history of fever in the family, history of anti-malaria treatment used, history of blood transfusions and use of malaria vector control (mosquito nets, larviciding, indoor residual house-spraying). Every household member was asked to provide a finger prick blood sample for microscopic examination of malaria parasites.

Each village location was extracted using combinations of Google Earth and maps provided by the Provincial General Directorate of Health Affairs. The location of the household was used to compute risk factors such as distance from road networks, distance to the PHCC, distance from dams and rivers.

### Cross-sectional surveys

Population based, cross-sectional, active malaria case detection surveys were conducted in the three PHCCs areas. Two surveys were performed before the onset of malaria transmission seasons in May-July 2006 and May-July 2007, and two during the transmission seasons August–December 2006 and September-November 2007. The sample selection was based on the distance from the relevant PHCC. Four villages were randomly selected by balloting from the list of each Centre’s catchment villages, selecting two villages at a distance >5 km and two villages within a distance <5 km from each PHCC. The 12 villages selected for the four cross-sectional surveys are shown in (Figure 1). Based on the total population size in the study area, a sample size of 3623 individuals was calculated to be able to detect a 1% prevalence with 80% power and 5% precision. In each village all individuals were enumerated, pregnant women and individuals who had taken anti-malarial treatment in the week preceding were excluded. Verbal informed consent was obtained from each participant following an explanation of the survey purpose. Each household member provided a finger prick blood sample used to detect malaria parasites using a rapid test for antigen detection; Malaria combo test kit, a rapid test for multiple species antigens based on detection of histidine-rich protein II (PfHRP-II)(TECO malaria combo test, Anaheim, CA 92807, USA). In addition, any history of illness, symptoms and body temperatures were recorded. Surveys and blood sampling were undertaken by health workers trained to interpret the test results in the field and administer treatment.

### Data entry and statistical analysis

Data were entered and analyzed using simple descriptive frequencies using EPI-INFO version 6.0.

### Ethical approval

Ethical approval was provided by Aseer General Directorate of Health Affairs (50/38/721) for the community cross-sectional surveys, and verbal consent was obtained from each participant, all data remained anonymous and linked only to subject numbers.

## Results

### Passive case detection (PCD)

A total of 1840 febrile patients suspected of malaria were screened between March 2006 and August 2007 at the three PHCs. The majority of the screened patients came from Alfarsha PHCC (60%), 30% were screened at Wadi Alhayah PHCC, and only 10% were screened from Albuqaa PHCC (10%). Among those screened older children (37%) and adults (37%) were more likely to present with fever and symptoms suggestive of malaria than younger children (26%).

**Figure 2:**
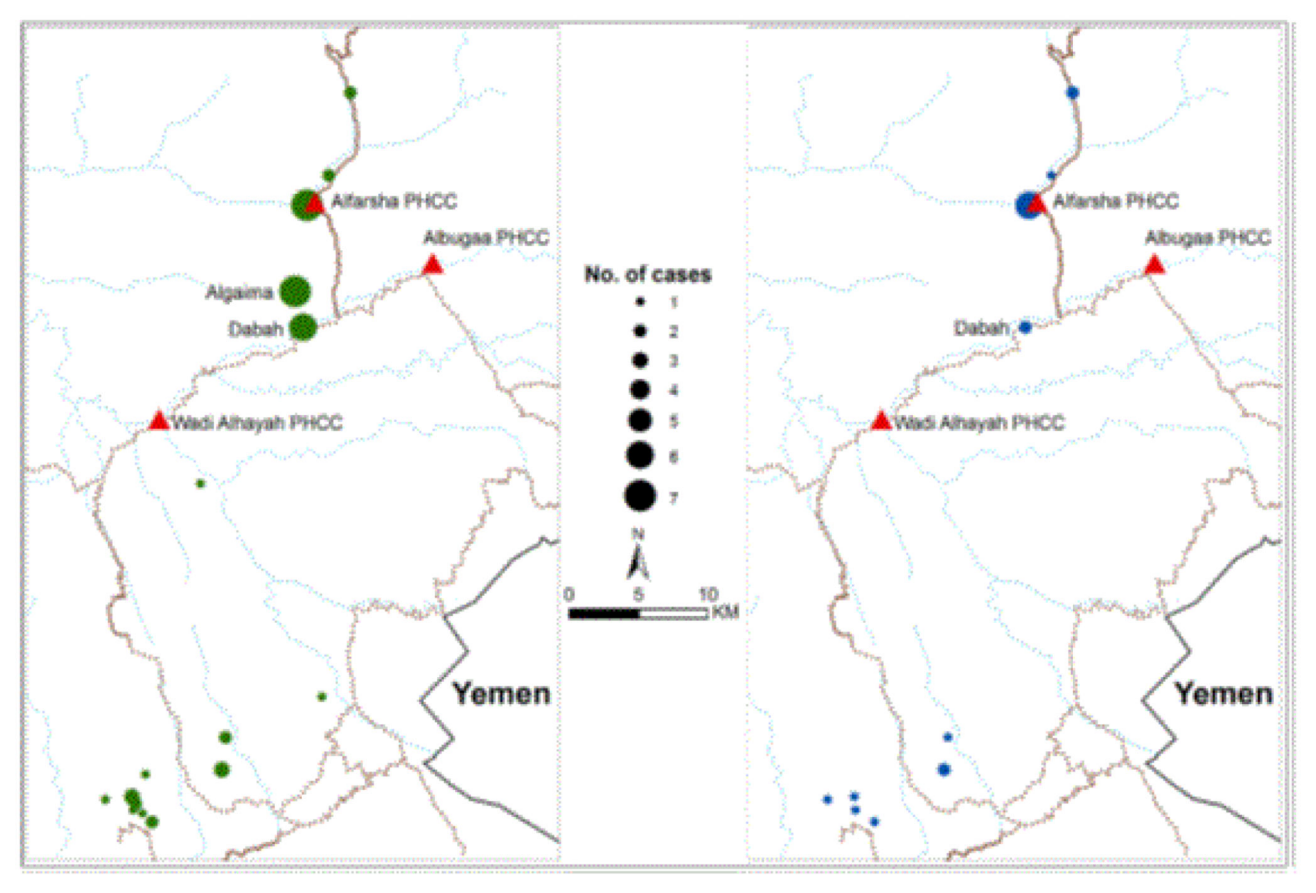
Map showing the location of 49 cases of malaria detected in 2006-2007 Green dots left hand side and the 18 cases of malaria without history of travel, blue dots on right hand side.

Only 49 (2.7%) cases of malaria infection were detected among those screened, 48 *P. falciparum* infections and one case of *P. vivax* detected at Wadi Alhaya PHCC. The majority of cases (34) were detected at Alfarsha PHCC resulting in an overall slide positivity rate of 3.1% at this PHCC. Across all PHCCs, infections were detected in all age groups, however they were more common in males and those aged above 15 years (Table 1). Cases were predominantly of Saudi origin (Table 1). 31 (63%) had travelled to, or from, malarious areas in the last two weeks. These infections are all likely to have been imported infections, most notably the *P. vivax* infection recorded in an Indian male recently arrived from his native country. Only 18 infections were detected among individuals who had not travelled in the last two weeks, and were most likely to be infections locally acquired. Among all infections 39 had reported sleeping outside, 42 were located in areas where illegal laborers were located either within the homestead or in close proximity (Table 1). The location of presumed imported and locally acquired cases is shown in Figure 2. In the majority of residential homes where cases lived, indoor residual house-spraying was reported to be high (74%) and the reported use of insecticide treated nets was 34% (Table 1). Seven of the detected infections were residents of the village where the PHCC Alfarsha was located, and seven cases were detected at Algaima village and six at Dabha village, in close proximity to Alfarsha PHCC and representing the largest clusters of cases. In addition, there were clusters of cases close to the border with Jazan region in the south (Figure 2). A similar pattern was observed for the 18 infected patients who had reported no recent travel (Figure 2).

### Community survey active case detection (ACD)

A total of 3623 individuals were screened using an RDT during four cross-sectional surveys in 12 villages surrounding each of the three PHCCs. Children of less than 5 years accounted for 11.4% of the examined population, and the mean age of the entire sample was 16.6 years (range, 1-70 years). 92% of those sampled were Saudi nationals. No positive RDT result was recorded during the examination of 1756 individuals during the low transmission seasons. During the high transmission season 16 (0.9%) RDT positive individuals were found to have a positive RDT for *P. falciparum*. From brief histories taken at the time of survey, none of these infected individuals had travelled in the last two weeks. The overall malaria prevalence rate across all four surveys was 0.44%.

## Discussion

The present study is the first to describe in detail the characteristics of malaria cases detected through the PCD system and the first cross-sectional survey of infection prevalence for thirty years in the Tihama Qahtan area. From the combined findings of the cross-sectional surveys and the PCD investigations, is evident that local transmission of *P. falciparum* was occurring during 2006-2007, albeit at very low levels. Malaria infection rates among febrile patients presenting to clinics was only 2.7%, however only 18 of these 49 infections were likely to have been the direct result of local transmission. Defining the location of infections versus the residence of detected cases is complex [19]. It was not possible to distinguish between local infections and imported infections in this study. While it is not possible to undertake any formal analysis of clustering of infection risks, it was notable that many of the smaller clusters of residences of infected patients were along the border with Jazan region (Figure 2). The focus of the present analysis has been on characterizing infected patients during 2006 and 2007. Travel, being a non-resident, and proximity to illegal migrants was associated with the majority of detected infections. Thus importation represents a sustained source of external, infection into the Tihama Qahtan area. Increased awareness is required regarding the threats posed by travel, both for providers of diagnostic services and the community at large. Illegal laborers from malaria-endemic areas of Yemen increase during the rainy seasons, when travelling by foot is more tolerable than during the hot dry season, as such they enter the Kingdom at times of peak of vector density. Migrant labor poses a complex problem. Screening of formal and informal workers, while politically and socially sensitive, might serve as a useful means to remove the consequences of imported infections.

Among the 18 infections likely to have been acquired within the area, the majority were males and sleeping outdoors was mentioned a normal behavior. This is important because the vector in this area, *An. arabiensis*, is exophilic, biting outdoors in the early to late evening. During the main transmission season night time temperatures can rise to as high as 30°C [20] forcing people to sleep outside. This has implications for risks from infected vectors and the effective use of insecticide treated nets and indoor residual house-spraying.

More effective might be to target larval stages of vectors, which is current practice by the Aseer Vector Borne Disease Administration.

Infection prevalence, as one might expect, was lower among those seen during active case detection in 12 villages that surround the three PHCCs. Across four surveys, and 3623 person observations, only 16 positive RDT results were recorded, all positives were from 10 of the 12 villages and only detected during the main transmission seasons. All infections were *P. falciparum*. Despite being a traditionally stable endemic area for *P. falciparum* transmission, and a major public health threat, by 2006/2007, local transmission had reached very low levels. Importantly, none of the 16 infections detected on cross-section were symptomatic at the time of the survey. While residual infections can be detected with RDTs, these asymptomatic carriers might continue to contribute to malaria transmission unless radically treated. It is not clear whether these individuals would have become symptomatic in the days after the survey had they not been treated or would have remained asymptomatic. If the latter this would pose a continued problem when aiming to identify and remove the last active residual foci of transmission [19].

The epidemiology of malaria in Tihama Qahtan, has changed considerably over the last thirty years. Reasons for the broader decline in malaria incidence across Aseer will be examined elsewhere (Al Sharani et al., in preparation), and likely to be a result of increased investment in malaria control operations since 2004, improved living standards, housing structures and increased networks of PHCC facilities and roads. By 2007, residual transmission was still present and the purpose of the present study was to interrogate factors likely to contribute to the locally acquired infections. Such investigations require minimal additional data, organized here as a study, but possible to integrate into routine PCD and ACD systems. Further analysis might, in future, be possible where adequate “controls” could be simultaneously defined, for example febrile, patients from the same geographic area, week of detection but without the evidence of malaria infection. This would provide opportunities to under-take a more formal risk factor analysis within a case-control framework [21]. In addition, it might be necessary to confirm any residual asymptomatic infections with more sensitive parasite detection methods such as polymerase chain reaction investigations from mass blood surveys [22].

## Conclusions

Local malaria transmission was evident in the Tihama Qahtan area in 2006-2007, however prevalence and incidence of new infections was very low, making the future ambitions of elimination biologically feasible. The constant source of imported infections must be considered in the area’s elimination ambitions, alongside strong behavioural community messages about sleeping outdoors unprotected and travel to malaria endemic areas outside the province. Being able to distinguish the origin of new infections in the area is a critical step to defining the basis of foci targeting of intervention during the elimination phase.

## Figures and Tables

**Table 1:** Characteristics of 49 cases of malaria detected at three PHCC in Tihama Qahtan March 2006-August 2007.

Characteristics	Frequency
Males	38 (77.6%)
Age	
>15 years	29 (59.3%)
5-14 years	11 (22.4%)
1-4 years	6 (12.2%)
<1 year	3 (6.1%)
Saudi resident	34 (87.8%)
Slept outdoors	39 (79.6%)
Reported use of a mosquito net	17 (34.7%)
Reported house being sprayed during previous 6 months	36 (73.5%)
Travelled in last 2 weeks to malaria area	31 (63.3%)
Occupation	
Clerk	12 (24.5%)
Housekeeper	6 (12.2%)
No formal work	31 (63.3%)
Housing type	
Mud	14 (28.6%)
Stone	28 (57.1%)
Concrete	7 (14.3%)
Proximity to illegal migrants	42 (85.7%)
More than 2 km from road	34 (69.4%)
More than 5 km from nearest PHCC or hospital	36 (73.5%)
Located within 2 km of a dam	49 (100%)
Located within 2 km of a seasonal river	49 (100%)
